# Serpentinization-Influenced Groundwater Harbors Extremely Low Diversity Microbial Communities Adapted to High pH

**DOI:** 10.3389/fmicb.2017.00308

**Published:** 2017-03-01

**Authors:** Katrina I. Twing, William J. Brazelton, Michael D. Y. Kubo, Alex J. Hyer, Dawn Cardace, Tori M. Hoehler, Tom M. McCollom, Matthew O. Schrenk

**Affiliations:** ^1^Department of Microbiology and Molecular Genetics, Michigan State University, East LansingMI, USA; ^2^Department of Biology, University of Utah, Salt Lake CityUT, USA; ^3^SETI Institute, Mountain ViewCA, USA; ^4^Department of Geosciences, University of Rhode Island, KingstonRI, USA; ^5^Exobiology Branch, NASA Ames Research Center, Moffett FieldCA, USA; ^6^Laboratory for Atmospheric and Space Physics, University of Colorado, BoulderCO, USA

**Keywords:** serpentinization, alkaliphile, extremophile, groundwater, borehole

## Abstract

Serpentinization is a widespread geochemical process associated with aqueous alteration of ultramafic rocks that produces abundant reductants (H_2_ and CH_4_) for life to exploit, but also potentially challenging conditions, including high pH, limited availability of terminal electron acceptors, and low concentrations of inorganic carbon. As a consequence, past studies of serpentinites have reported low cellular abundances and limited microbial diversity. Establishment of the Coast Range Ophiolite Microbial Observatory (California, U.S.A.) allowed a comparison of microbial communities and physicochemical parameters directly within serpentinization-influenced subsurface aquifers. Samples collected from seven wells were subjected to a range of analyses, including solute and gas chemistry, microbial diversity by 16S rRNA gene sequencing, and metabolic potential by shotgun metagenomics, in an attempt to elucidate what factors drive microbial activities in serpentinite habitats. This study describes the first comprehensive interdisciplinary analysis of microbial communities in hyperalkaline groundwater directly accessed by boreholes into serpentinite rocks. Several environmental factors, including pH, methane, and carbon monoxide, were strongly associated with the predominant subsurface microbial communities. A single operational taxonomic unit (OTU) of Betaproteobacteria and a few OTUs of Clostridia were the almost exclusive inhabitants of fluids exhibiting the most serpentinized character. Metagenomes from these extreme samples contained abundant sequences encoding proteins associated with hydrogen metabolism, carbon monoxide oxidation, carbon fixation, and acetogenesis. Metabolic pathways encoded by Clostridia and Betaproteobacteria, in particular, are likely to play important roles in the ecosystems of serpentinizing groundwater. These data provide a basis for further biogeochemical studies of key processes in serpentinite subsurface environments.

## Introduction

The Earth’s subsurface is predicted to be an expansive habitat for microorganisms (Whitman et al., 1998; Edwards et al., 2012; Kallmeyer et al., 2012). Unlike surface biomes, the subsurface is largely decoupled from photosynthetic primary production; instead, many subsurface ecosystems are influenced by carbon and energy liberated from the Earth’s mantle and crust. However, given the inherent lack of accessibility, direct sampling of subsurface, rock-hosted environments has been limited. In continental settings, researchers have used caves (Northrup and Lavoie, 2001; Engel et al., 2004), mines (Onstott et al., 2003; Chivian et al., 2008), springs (Brazelton et al., 2012, 2013; Suzuki et al., 2013; Cardace et al., 2015), and isolated boreholes (Stevens and McKinley, 1995; Itävaara et al., 2011) as windows into the subsurface environment. These features grant access to an otherwise inaccessible environment, but they represent opportunistic sampling locations. In the present study, a series of wells were drilled directly into serpentinization-influenced aquifers of the Coast Range Ophiolite, a portion of ancient seafloor in northern California, USA, to sample microbial communities in serpentinizing rocks and groundwater. This observatory represents the first opportunity to investigate microbial communities with direct access to the range of conditions in the serpentinizing subsurface (Cardace et al., 2013).

Serpentinization is a widespread geochemical process involving the aqueous alteration of peridotite to serpentine minerals, resulting in an abundance of potential reductants, in the form of hydrogen, methane, and small organic molecules (McCollom and Seewald, 2007; Proskurowski et al., 2008; Schrenk et al., 2013). Serpentinization also releases hydroxyl ions, which creates extremely high pH fluids (pH > 10). At high pH, bicarbonate and carbonate are the dominant species of dissolved inorganic carbon (DIC), and the latter can precipitate out of solution as carbonate minerals when in the presence of divalent cations, such as Ca^2+^ and Mg^2+^ commonly found in serpentinite fluids. Thus, fluids associated with serpentinization are characteristically low in DIC, particularly dissolved CO_2_. Compared to the abundance of reductants in these systems, there is a lack of corresponding oxidants, which likely limits the range of potential microbial metabolisms. Thus subsurface serpentinite environments are characterized by unusual challenges to life, such as extreme pH (>10), limited availability of dissolved carbon, and a lack of potential terminal electron acceptors.

The best-characterized serpentinite-hosted microbial ecosystem to date is the Lost City Hydrothermal Field, located 15 km from the Mid-Atlantic Ridge (Kelley et al., 2005). The tall carbonate chimneys at Lost City are dominated by methane-cycling archaea in the anoxic chimney interiors (Schrenk et al., 2004) and by methanotrophic and sulfur-oxidizing bacteria in the chimney exteriors (Brazelton et al., 2006). More recently, researchers have started exploring life within continental serpentinite environments by using natural springs, such as the Tablelands Ophiolite in Newfoundland, Canada (Brazelton et al., 2012, 2013) and The Cedars site in northern California (Suzuki et al., 2013, 2014), or previously established wells, such as at the Cabeço de Vide Aquifer (CVA) in Portugal (Tiago and Veríssimo, 2013). In these studies of continental serpentinite sites, microbial communities were dominated by clades of Betaproteobacteria and Firmicutes (Schrenk et al., 2013).

Surveys of the Tablelands Ophiolite suggest that subsurface serpentinite communities are dominated by Erysipelotrichia, a class within the phylum Firmicutes, in the deep, anoxic source-waters and microaerophilic H_2_-oxidizing Betaproteobacteria at the shallow, oxic/anoxic interface (Brazelton et al., 2013). Microcosm experiments from the Coast Range Ophiolite Microbial Observatory (CROMO), the location of this study, have indicated that Betaproteobacteria closely related to *Hydrogenophaga pseudoflava* and Clostridia (phylum Firmicutes) closely related to *Dethiobacter alkaliphilus*, are stimulated by small organic molecules that are expected to be available in the serpentinite environment (Crespo-Medina et al., 2014). Furthermore, recently published genomes of cultivated isolates of the proposed genus *Serpentinomonas*, which are most closely related to the genus *Hydrogenophaga*, are consistent with a role for these organisms at oxic/anoxic interfaces in serpentinizing systems (Suzuki et al., 2014).

While these previous studies suggest that distinct microbial communities inhabit different physico-chemical regimes in serpentinizing groundwater, these relationships have not yet been studied directly. Furthermore, little genomic or metagenomic data for organisms other than *Serpentinomonas* are available from serpentinizing environments. This study combines environmental 16S rRNA gene sequencing, shotgun metagenomic analyses, and geochemical monitoring across a range of conditions in order to relate patterns in microbial diversity and metabolic potential to underlying geochemical processes in serpentinite subsurface environments. This work improves our understanding of the physiology and ecology of the dominant bacteria in these ubiquitous ecosystems, and it will facilitate our integration of these systems into models of carbon cycling.

## Materials and Methods

### Site Description and Sample Collection

The Coast Range Ophiolite is a 155–170 million year old ophiolite located in northern California, containing numerous calcium-hydroxide rich springs, indicating serpentinizing activity below the surface (Barnes and O’Neil, 1971). The CROMO, which is located at the UC-Davis McLaughlin Natural Reserve in Lower Lake, CA and was established in August 2011 and using clean drilling techniques to enable subsequent monitoring of the microbial communities and associated geochemistry within the serpentinite subsurface (Cardace et al., 2013). CROMO consists of two sets of wells located 1.4 km apart: the Core Shed Wells (CSW), and the Quarry Valley wells (QV). CSW consists of five wells, drilled to depths between 9 and 31 m. QV consists of three wells, drilled to depths between 15 and 46 m.

Preliminary lithostratigraphic interpretations of CROMO cores indicate that both sites (CSW and QV) are characterized by intercalated serpentine-rich units with variable contributions of other clay minerals; lizardite and magnetite are common in serpentine-rich units (Cardace et al., 2013). At specific intervals, minerals indicative of altered mafic rocks (e.g., albite, chlorite, quartz, rarely calcite) co-occur with serpentine minerals, such as at ∼28 m depth at the primary CSW site (CSW1.1), and at 18–22 m and 34–36 m depth at the primary QV site (QV1.1). Very thin serpentine-rich soil cover exists at the QV1.1 site (<1 m), while ∼4 m of soil cover occurs at CSW1.1 (Cardace et al., 2013). Taken together, these data indicate that CROMO scientific monitoring wells sample fluids interacting with tectonically reworked ultramafic units very near the surface, with some entrainment of altered mafic materials from adjacent units of the Coast Range Ophiolite.

The samples described here were collected from seven wells at CROMO in August 2012. For the current study, well QV1.3 was not sampled due to complications with sediments clogging the filters. Well fluids were collected using positive displacement Teflon bladder pumps (Geotech Environmental Equipment, Denver, CO, USA) and pumped through a YSI 3059 flow cell fitted with a YSI 556 multiprobe (Yellowsprings, OH, USA), which measured water temperature, specific conductance, pH, dissolved oxygen (DO) and oxidation-reduction potential (ORP) once the DO measurement stabilized at a minimum value. Samples were collected for dissolved gas analyses (CH_4_, CO, and H_2_) and aqueous phase species (DIC and organic acids), as previously described Crespo-Medina et al. (2014).

For DNA analyses, fluids were filtered through a 0.22 μm Sterivex filter cartridge (Millipore, Billerica, MA, USA) using a Masterflex E/S peristaltic pump (Cole Parmer, Vernon Hills, IL, USA). Field replicate samples, ranging between two to eight filters per well, were collected in succession (labeled A, B, C, etc.). Sterivex filter cartridges were flash frozen with liquid nitrogen and stored at -80°C until DNA extraction. For microbial cell quantification, replicate samples of 45 mL of fluids were preserved at a final concentration of 3.7% formaldehyde and stored at 4°C. All publicly available data generated from this project can be found^1^.

### Geochemistry

Dissolved gasses (H_2_, CH_4_, and CO) were extracted into an inert (N_2_) gas phase of known volume and analyzed for CH_4_ via a SRI 8610C GC-FID and dissolved H_2_ and CO with a Trace Analytical RGA3 Reduced Gas Analyzer. DIC was measured by acidifying a known volume of well fluid within a sealed vial, and analyzing the concentration of liberated CO_2_ in the headspace by GC-FID (SRI 8610) following passage through a “methanizer,” which catalyzes the in-line conversion of CO and CO_2_ to methane in the presence of H_2_ over a heated Ni catalyst, thus allowing sensitive detection of these species by flame ionization detector following their separation by gas chromatography. Organic acid samples were analyzed by HPLC with UV/VIS detection, following derivatization with 2-nitrophenylhydrazide (Albert and Martens, 1997). All sample vials were analyzed with duplicate injections.

### Microbial Cell Counts

Fluids preserved for cell counts were filtered through 0.2 μm black polycarbonate filters (Millipore, Billerica, MA, USA). The cells were stained with 1 μg/ml of 4′,6-diamidino-2-phenylindole (DAPI) and were counted by epifluorescence microscopy using appropriate filter sets according to previously published protocols (Hobbie et al., 1977; Schrenk et al., 2003).

### DNA Extraction

DNA extractions from Sterivex filter cartridges were performed by lysis via freeze/thaw cycles and lysozyme/Proteinase K treatment and purified with phenol-chloroform extractions, precipitation in ethanol, and further purification with QiaAmp (Qiagen, Hilden, Germany) columns according to the manufacturer’s instructions for purification of genomic DNA, as described previously by Brazelton et al. (2013).

### 16S rRNA Gene Amplicon Sequencing and Data Analysis

Samples were submitted to the DOE Joint Genome Institute (JGI) for 16S rRNA amplicon sequencing of the V4 region on an Illumina MiSeq instrument, as described by Caporaso et al. (2011). Briefly, the amplification reaction contained 5 PRIME’s HotMasterMix, custom V4 16S rRNA gene primers, and Illumina sequencing adapters and unique barcodes. The individual amplicon libraries were quantified, normalized, and pooled. The pooled multiplex reactions were then quantified using KAPA Biosystem’s next-generation sequencing library qPCR kit and run on a Roche LightCycler 480 real-time PCR instrument. The quantified, multiplexed amplicon pool was then loaded on an Illumina MiSeq instrument utilizing the v3 reagent mix and a 2 × 300 indexed recipe mix.

Sequence reads were aligned to the SILVA SSURef alignment (v119), and taxonomic classifications were assigned using mothur (Pruesse et al., 2007; Schloss et al., 2009). Sequences were clustered into operational taxonomic units (OTUs) at the 3% distance threshold using the cluster.split command and the average-neighbor clustering algorithm in mothur (Schloss and Westcott, 2011). Prior to calculating measures of diversity, data were subsampled to the sample with the fewest sequences (77,580). Beta (between sample) diversity of the microbial communities was assessed by calculation of the Bray–Curtis index and displayed in a multi-dimension scaling (MDS) plot with geochemical data overlay in Primer-6 (Clarke, 1993; Clarke and Gorley, 2006). Alpha (within sample) diversity was assessed with the Inverse Simpson diversity index and rarefaction analysis. Sequence identification of reads belonging to the top OTUs compared with 16S rRNA sequences from other serpentinite studies (Brazelton et al., 2013; Suzuki et al., 2013; Tiago and Veríssimo, 2013) was performed using MatGAT with the default settings (Campanella et al., 2003). The 16S rRNA sequence data are publicly available in the NCBI Sequence Read Archive under the accession number SRA280854.

### Metagenomic Sequencing and Data Analysis

Samples were submitted to JGI for metagenomic sequencing on an Illumina HiSeq2000 instrument, as described by Hawley et al. (2014). Briefly, 200 ng of DNA was used for each sample and sheared to 270 bp fragments via a Corvaris LE220 focused-ultrasonicator and size selected by SPRI. Fragments were then end-repaired, A-tailed, and ligated with Illumina-compatible adapters with barcodes unique for each library. Libraries were quantified with KAPA Biosystem’s next-generation sequencing library qPCR kit and run on a Roche LightCycler 280 real-time PCR instrument. Quantified libraries were combined into 10-library pools and prepared for sequencing on the Illumina instrument in one lane each, using the TruSeq paired-end cluster kit (v3) and Illumina’s cBot instrument to generate clustered flowcells, which were sequenced on the Illumina HiSeq2000 sequencer using TruSeq SBS sequencing kit v3 and a 2 × 150 indexed run recipe.

Metagenomic assembly was conducted by JGI as described by Hawley et al. (2014) and briefly described again here. Raw reads were trimmed with a minimum quality score cutoff of 10, and the trimmed paired-end reads were assembled with SOAPdenovo v1.05, with the default settings and a variety of kmers (i.e., 81, 85, 89, 93, 97, 101; Luo et al., 2012; Hawley et al., 2014). Contigs were sorted into pools based on length: contigs < 1800 bp were further assembled by Newbler (Life Technologies, Carlsbad, CA, USA) and contigs > 1800 bp, including those produced from Newbler run, were combined using minimus 2 (flags: -D MINID = 89 –D OVERLAP = 80; Sommer et al., 2007). BWA was used to estimate read depth, based on mapping of trimmed, screened, paired-end Illumina reads to assembled contigs (Li and Durbin, 2009). These data are publicly available in the JGI IMG/M database^2^ under the project IDs: 1021918, 1021921, 1021924, and 1021927; and in the MG-RAST database (Meyer et al., 2008) under the following sample IDs: 4569549.3, 4569550.3, 4569551.3, and 4569552.3.

The Prokka pipeline (Seeman, 2014) was used for gene prediction and functional annotation of contigs. The arguments –metagenome and –proteins were used with Prokka v.1.12 to indicate that genes should be predicted with the implementation of Prodigal v.2.6.2 (Hyatt et al., 2010) optimized for metagenomes. Predicted protein-coding sequences were aligned to the last free version (2011) of the Kyoto Encyclopedia of Genes and Genomes (Ogata et al., 1999) using BLASTP v2.3, and any coding sequences that remained un-annotated were then aligned to Prokka’s default databases. Predicted protein abundances (in units of reads per kilobase) were calculated with HTSeq v.0.6.1 (Anders et al., 2014), and the final normalized coverage was calculated by normalizing to the total number of bases in the smallest metagenome.

To assign taxonomy to specific contigs of interest, a consensus taxonomic classification was manually determined by examining the taxonomic classifications associated with the best BLAST hits for each predicted gene. Specifically, for each contig-of-interest, all (Prokka-predicted) coding sequences (CDSs) on the contig were aligned against the NCBI NR database (v. 2016-10-01), and the taxonomy of the best hit was taken to be the taxonomy of the CDS. The taxonomy of the whole contig was manually determined by identifying the lowest common ancestor on the NCBI Taxonomy Tree where half of all CDS taxonomic assignments agreed (Hanson et al., 2016). The full list of CDS taxonomic assignments can be found in Supplementary Dataset S1.

### Statistical Analyses

Correlation network analyses were constructed from statistically significant pairwise Pearson’s correlations among environmental variables and sequence data (Fuhrman and Steele, 2008) and visualized in Cytoscape v 2.8.3 (Shannon et al., 2003). A matrix containing environmental data and relative OTU (97% similarity) abundance for each sample was used as input for pairwise Pearson’s correlation analysis computed with the rcor.test function in the R package lmt (Rizopoulos, 2006). The false-discovery rate (*q*-value) was computed for the distribution of Pearson’s *p*-values to account for multiple tests. Pairwise correlations with both *p*- and *q*-values of <0.05 were considered significant and included in network analyses. Network models of significant correlations were created using Cytoscape v2.8.3 (Shannon et al., 2003).

The ANOSIM test using a Bray–Curtis resemblance matrix, with sequence data subsampled to 77,580 sequences (the size of the smallest 16S rRNA amplicon library), was used to test whether individual environmental parameter categories had significant effects on the community composition of samples (Clarke, 1993). To statistically determine which combinations of numerical environmental variables best described the community composition variation within the dataset, the BEST analysis was performed in PRIMER-6 (Clarke, 1993; Clarke and Gorley, 2006).

## Results

### Sampling Site and Geochemistry

Fluids were collected from seven wells within the CROMO, which were drilled for the purpose of monitoring biogeochemistry and microbial community dynamics with high temporal and spatial resolution (Cardace et al., 2013). To identify which bacterial taxa are most influenced by geochemical indicators of serpentinization, geochemical and microbiological data from these seven wells were compared.

Geochemical data associated with the well fluids from August 2012 are summarized in **Table 1**. Samples from wells CSW1.1 and QV1.1 are characterized by extremely high pH (12.2 and 11.5, respectively) and generally reducing character (**Table 1**). These wells are depleted in DIC, containing one to two orders of magnitude less DIC than a nearby well with circumneutral pH, CSW1.4 (**Table 1**). CSW1.1 also had higher concentrations of H_2_ and organic acids, relative to the other wells (**Table 1**). QV1.1, the deepest well, contained the highest cell abundance and exhibited the lowest dissolved O_2_ measurement. Wells CSW1.4 and QV1.2 exhibited circumneutral pH, low conductivity and higher DIC (**Table 1**) than the other wells. These circumneutral pH wells also had elevated concentrations of H_2_ and CO comparable to the wells with higher pH. CSW1.2 (pH 9.3) had the highest concentration of methane (1.6 mM; **Table 1**).

**Table 1:** Environmental and geochemical parameters associated with samples collected in August 2012.

	CSW1.1	CSW1.2	CSW1.3	CSW1.4	CSW1.5	QV1.1	QV1.2
Depth (mbs)	31.1	19.2	23.2	8.8	27.4	45.7	14.9
Temp (°C)	17.2	18.5	16.9	15.2	16.2	17.9	18.4
pH	12.2	9.3	10.1	7.9	9.7	11.5	7.9
ORP (mV)	-284	-32	-83	-35	-121	-155	-30
DO (mg/L)	0.05	0.41	0.06	1.05	0.03	0.03	0.03
Conductivity (μS/cm)	5200	3710	4500	1560	4220	2068	1655
DIC (μM)	253 ± 8	605 ± 268	172 ± 16	5046 ± 531	545 ± 13	96 ± 2	979 ± 32
Dissolved H_2_ (μM)	0.289 ± 0.004	0.140 ± 0.001	0.283 ± 0.018	0.271 ± 0.013	0.138 ± 0.020	0.075 ± 0.001	0.076 ± 0.009
Dissolved CH_4_ (mM)	0.524 ± 0.132	1.625 ± 0.055	0.969 ± 0.529	0.002 ± 0.0003	1.266 ± 0.032	0.301 ± 0.021	0.303 ± 0.030
Dissolved CO (μM)	0.089 ± 0.002	0.158 ± 0.001	0.115 ± 0.013	0.187 ± 0.005	0.124 ± 0.008	0.142 ± 0.004	0.150 ± 0.008
Acetate (μM)	70.79 ± 1.26	<1.55	<1.55	<3.04	<3.04	10.20 ± 0.33	<2.01
Formate (μM)	15.74 ± 0.99	<1.39	<1.39	<1.79	<1.79	<1.39	<2.23
Propionate (μM)	3.49 ± 0.003	<0.01	<0.01	<0.01	<0.01	0.16 ± 0.05	0.22 ± 0.01
Butyrate (μM)	20.99 ± 0.45	<1.11	<1.11	<2.75	<2.75	5.97 ± 0.30	<1.89
Microbial cells (cells/mL)	1.8 × 10^5^	6.6 × 10^5^	2.3 × 10^5^	1.0 × 10^5^	3.9 × 10^5^	1.0 × 10^6^	9.5 × 10^5^

*Values reported are averages of replicate measurements taken throughout sampling. Specific parameter values associated with specific field replicate samples can be found in **Supplementary Figure S2**.*

Unsurprisingly, many of the environmental parameters of the system (**Table 1**) were correlated with one another (Supplementary Table S1). Higher pH was correlated with more negative ORP, and lower concentrations of carbon monoxide (CO), DO, and DIC, and was positively correlated with depth and organic acid concentration (Supplementary Table S1). The concentrations of the organic acids acetate, formate, propionate, and butyrate were all positively correlated with one another and were negatively correlated with ORP (Supplementary Table S1). CO concentration was positively correlated with ORP (i.e., positively correlated with a more positive ORP value) and DO, and was negatively correlated with conductivity and H_2_ concentration (Supplementary Table S1). The concentration of methane was not significantly correlated with any other environmental parameters.

### 16S rRNA Gene Diversity and Community Composition

Bacterial diversity was assessed in fluids collected in August 2012 from the seven CROMO wells. Environmental sequences of 16S rRNA gene amplicons were obtained with an Illumina MiSeq platform, yielding between 78,000 and 179,000 merged paired-end sequences per sample, for a total of 2,528,572 16S rRNA sequences in this study. These sequences were clustered into 11,454 OTUs at a 97% sequence similarity threshold, and only 30 of these OTUs comprised greater than 1% of the sequences in any of the samples analyzed. All diversity analyses in this study were conducted with OTUs, instead of relying solely on taxonomic annotations, in order to avoid the biases and limitations inherent to database-dependent classifications that are magnified when studying poorly characterized microbial communities. Field replicates of samples were collected and analyzed in parallel and were statistically indistinguishable from one another, as determined by a SIMPROF test of community similarities among all samples (**Supplementary Figure S1**). The community compositions of samples from different wells were clearly distinct from each other (ANOSIM, *R* = 0.96, *p*-value = 0.001).

**FIGURE 1:**
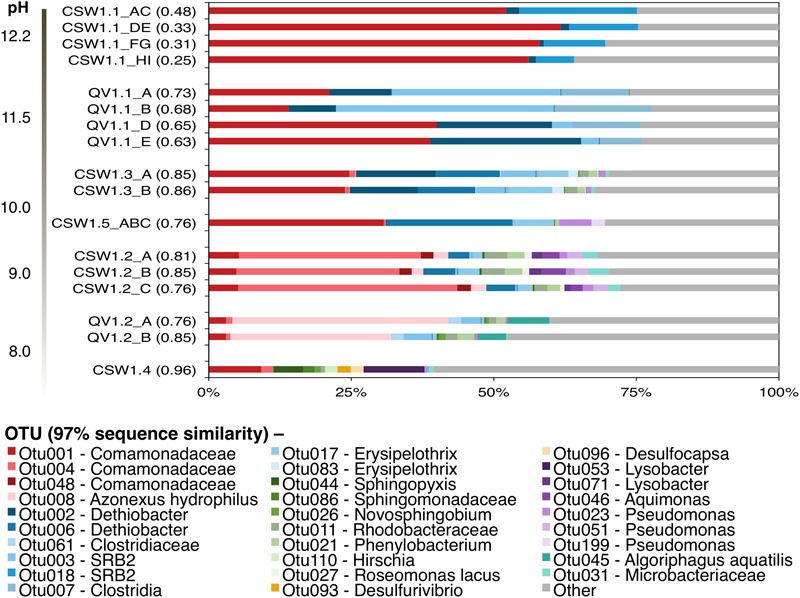
**Inter-well community composition comparison from August 2012.** OTUs were formed at the 97% similarity level in mothur using the average-neighbor algorithm. Bars indicate individual OTUs and are color-coded by Phlyum/Class: β-proteobacteria (red), Firmicutes (blue), α-proteobacteria (green), δ-proteobacteria (orange), γ-proteobacteria (purple), and Actinobacteria (teal). Other (gray bars) represents any OTUs making up <1% of all samples in the dataset. Samples are organized by pH (ANOSIM: *R* = 0.66, *p*-value < 0.05). Replicates represent field replicate samples collected in successive order on the day. The numbers in parentheses beside the sample names represent the Inverse Simpson diversity index.

Alpha diversity of the samples, as measured by the Inverse Simpson diversity index, decreased with increasing pH (**Figure 1**). The wells with the highest pH, CSW1.1 and QV1.1, exhibited extremely low diversity, containing almost exclusively Betaproteobacteria and Firmicutes (**Figure 1**). Well CSW1.1 was dominated by a single betaproteobacterial OTU (OTU001), classified as a member of family Comamonadaceae with 100% sequence identity over 250 bp of the 16S rRNA gene’s V4 region (calculated with MatGAT) to strain B1 from the proposed genus *Serpentinomonas* isolated from The Cedars serpentinite site (Suzuki et al., 2014; **Table 2**). The second most abundant OTU in CSW1.1 (OTU018), which comprised 12.5 ± 5.8% of the sequences from that well, was classified as Thermoanaerobacterales SRB-2, and exhibited 99% sequence identity to a Clostridia clone from a well in Cabeço de Vide (CVA) in Portugal (Tiago and Veríssimo, 2013; **Table 2**). The third most-abundant OTU detected in CSW1.1 (OTU002), which accounted for only 1% of the sequences from that well, was classified as *Dethiobacter*, and shared 100% sequence identity to a clone from CVA (Tiago and Veríssimo, 2013; **Table 2**), enriched in a microcosm from CROMO (Crespo-Medina et al., 2014), and a clone from the deep groundwater site at The Cedars (Suzuki et al., 2013). The remaining 29.0 ± 5.3% of the CSW1.1 microbial community was made up of rare species, defined as OTUs comprising less than 1% of the total sequences in any sample.

**Table 2:** Summary of significant correlations (*p*-value < 0.05) between top OTUs (making up >25% of the sample in which they are most abundant) and environmental parameters.

OTU	Variable	*R*	Corr^∗^	Max Sample	Max Abundance (% of sample)	Class	Order	Family	Closest Relative (NCBI accession number)	% Identity^†^
OTU001	Butyrate	0.88	+	CSW1.1	61.7	Betaproteobacteria	Burkholderiales	Comamonadaceae	*Serpentinomonas* B1 (AP014569.1)^a^	100%
	pH	0.86	+							
	Acetate	0.86	+							
	Propionate	0.83	+							
	Formate	0.82	+							
	Conductivity	0.63	+							
	Hydrogen	0.54	+							
	ORP	0.91	-							
	CO	0.86	-							
OTU004	Methane	0.81	+	CSW1.2	38.4	Betaproteobacteria	Burkholderiales	Comamonadaceae	*Alicycliphilus denitrificans*	100%
	DO	0.53	+						(NR_074585.1)^b^	
OTU003	Depth	0.54	+	QV1.1	38.2	Clostridia	Thermoanaerobacterales	SRB2	CVCloAm3Ph15 (AM778006)^c^	99.6%
OTU008	pH	0.61	-	QV1.2	38.0	Betaproteobacteria	Rhodocyclales	Rhodocyclaceae	*Azonexus Hydrophilus* (EF158391.1)^d^	100%
OTU002	Depth	0.67	+	QV1.1	26.5	Clostridia	Clostridiales	Syntrophomonadaceae	CVCloAm2Ph135 (AM777954)^c^	100%
OTU006	Methane	0.60	+	CSW1.5	21.9	Clostridia	Clostridiales	Syntrophomonadaceae	CVCloAm2Ph135 (AM777954)^c^	98.2%
OTU018	Formate	0.91	+	CSW1.1	20.6	Clostridia	Thermoanaerobacterales	SRB2	CVCloAm3Ph98	99.1%
	Propionate	0.91	+						(AM778028)^c^	
	Acetate	0.90	+							
	Butyrate	0.88	+							
	Conductivity	0.64	+							
	Hydrogen	0.60	+							
	pH	0.60	+							
	ORP	0.85	-							
	CO	0.74	-							

*^a^Suzuki et al., 2013; ^b^Oosterkamp et al., 2011; ^c^Tiago and Veríssimo, 2013; ^d^Chou et al., 2008. This is a subset of the data used to make the correlation network (**Figure 4**).*

*^∗^Corr, correlation relationship.*

*^†^Percent identity, as determined by MatGAT (Campanella et al., 2003).*

Well QV1.1 was dominated by three Clostridia OTUs (OTU003, OTU002, and OTU007) that together accounted for 47.1 ± 13.4% of the bacterial community (**Figure 1**). Both OTU003 (classified as Thermoanaerobacterales SRB-2) and OTU002 (classified as *Dethiobacter*) exhibited 99–100% sequence identity to clones from CVA in Portugal (Tiago and Veríssimo, 2013; **Table 2**). The same betaproteobacterial OTU from CSW1.1 (OTU001) made up 28.5 ± 12.9% of the QV1.1 community, and the remaining bacterial taxa were rare, accounting for 24.3 ± 1.6% of the community (**Figure 1**). A time-series analysis of samples collected quarterly from QV1.1 (data not shown) indicated that community composition within wells is relatively constant over time, with no significant difference between time points (ANOSIM, *R* = 0.2, *p*-value = 0.902).

While Betaproteobacteria made up a large proportion of all samples above neutral pH, the diversity and composition of the Betaproteobacteria shifted with pH (**Figure 1**). As expressed above, OTU001 made up 42.7 ± 17.6% of the extremely high pH wells. However, in samples with pH ≤ 10, OTU001 was replaced by OTU004 (classified as Comamonadaceae and 100% identical to *Alicycliphilus denitrificans*; **Table 2**) and OTU008 (classified as *Azonexus hydrophilus*; **Table 2**) as the dominant betaproteobacterial taxa.

Clostridia, which accounted for up to 64% of the bacteria in the highest pH fluids, were also found in the moderately high pH wells (defined here as wells with pH 8.5–10). *Dethiobacter* OTUs made up 13.9 ± 10.1% of samples with a pH 9.5–11.0. Erysipelotrichia (another class of the phylum Firmicutes) made up 8 and 2% of CSW1.5 (pH 9.7) and CSW1.2 (pH 9.3), respectively, but was not detected in any other CROMO samples. OTUs classified as Thermoanaerobacterales SRB-2 were detected in QV1.2, as well as CSW1.1 and QV1.1. No Firmicutes OTUs were detected in the wells with pH less than 9 (**Figure 1**). In addition to Betaproteobacteria and Firmicutes, the moderately high pH (pH 8.5–10) wells contained Bacteroidetes as well as Alpha-, Delta-, and Gammaproteobacteria (**Figure 1**). The circumneutral pH wells contained a greater complement of rare taxa and many taxa that were not present in the high pH wells (**Figure 1**).

**FIGURE 2:**
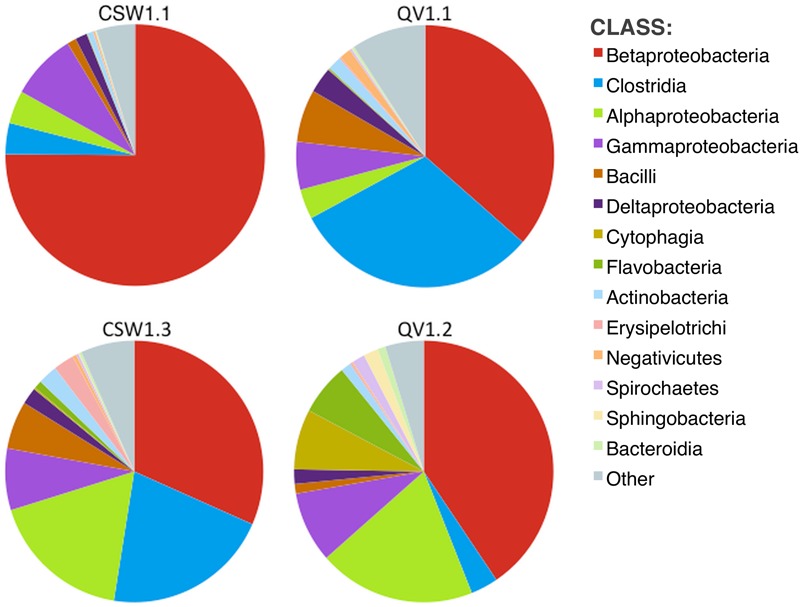
**Diversity (at class level) within metagenomic assemblies, as determined by MG-RAST (Meyer et al., 2008).** Other represents classes with relative abundance <1% in any sample. No archaeal classes comprised more than 1% of any sample.

No archaea were detected in any of the 16S rRNA amplicon libraries, which were created with the universal primers targeting the V4 region of the 16S rRNA gene used by the DOE Joint Genome Institute (Caporaso et al., 2011). In addition to 16S rRNA amplicon sequencing, a subset of the samples (CSW1.1AC, CSW1.3A, QV1.1A, and QV1.2A) underwent shotgun metagenomic sequencing. To further investigate the potential presence of archaea in CROMO fluids the relative abundance of archaea was also assessed by counting archaeal sequences in the metagenomic datasets. The number of metagenomic sequences classified as archaea by MG-RAST (Meyer et al., 2008) did not exceed 1% of the total sequences in any sample (**Table 3**). Furthermore, none of these archaeal metagenomic reads included a 16S rRNA gene. The bacterial diversity of the metagenomes exhibited similar abundances of Betaproteobacteria, but expressed a lower abundance of Clostridia and greater diversity of other taxa, compared to the 16S rRNA amplicon data (**Figures 1, 2**).

**Table 3:** Microbial communities at CROMO are dominated by Bacteria.

Well	Bacteria	Archaea	Eukaryotes	Viruses	Unassigned
CSW1.1	98.3	0.2	1.2	0.1	0.2
QV1.1	97.9	1.1	0.8	0.0	0.1
CSW1.3	98.8	0.7	0.3	0.0	0.1
QV1.2	99.1	0.2	0.5	0.1	0.1

*Relative abundance is expressed as percentage of metagenomic sequence reads assigned to different domains of life via the M5NR database in MG-RAST.*

### Biogeochemical Relationships

**FIGURE 3:**
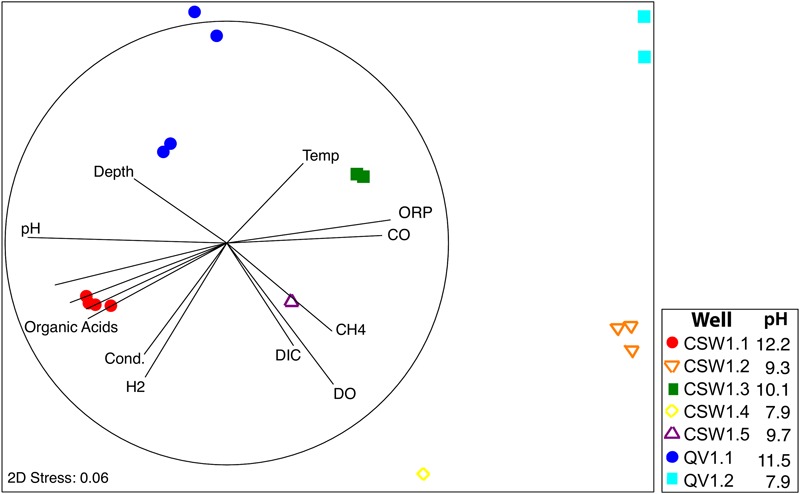
**Non-parametric Multi Dimensional Scaling (MDS) plot of microbial community diversity based upon bacterial 16S rRNA gene sequences using the Bray–Curtis similarity index.** Environmental variables that correlated with community composition are represented by vectors in two-dimensional space. The dark circle represents the length of a vector with perfect correlation (*R* = 1).

**FIGURE 4:**
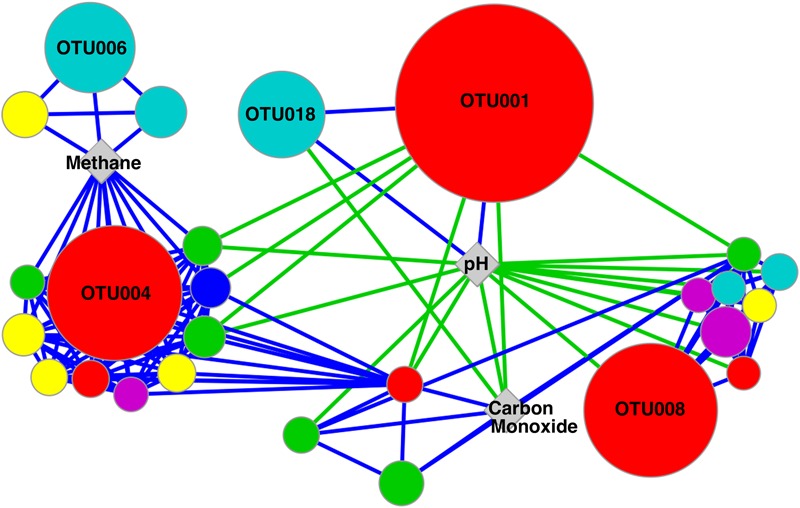
**Network diagram of significant correlations between OTUs and environmental variables identified in BEST analysis as accounting for 83% of the variation in community composition (*p*-value = 0.001).** OTU node size is relative to the maximum abundance of the OTU across the samples. Node color represents the taxonomic assignment of the OTU at the Phylum/Class level: β-proteobacteria (red), Firmicutes (blue), α-proteobacteria (green), γ-proteobacteria (teal), Actinobacteria (yellow), and Bacteroidetes (pink). Nodes represent OTUs with a relative abundance >1% of any sample, while OTUs making up >10% of any sample are labeled with OTU IDs. Positive and negative correlations are represented with blue and green lines, respectively.

One of the main goals of this study was to identify the geochemical drivers of microbial community composition within serpentinite subsurface environments. Several physical and chemical factors are overlain on a multi-dimensional scaling (MDS) plot of community composition (as measured by shared 16S rRNA OTUs; **Figure 3**). The figure highlights the geochemical variability among the wells and visually displays the correlated parameters (**Figure 3** and Supplementary Table S1). Community-level differences in the samples can be seen, such as a differentiation between high pH and moderate/circumneutral pH samples (**Figure 3**). A combination of pH, CO, and CH_4_ concentrations best explain the bacterial community composition variability across wells, as determined by the multivariate BEST test in Primer-6 ((*R* = 0.83, *p*-value = 0.001); Clarke, 1993; Clarke and Gorley, 2006). Therefore, pairwise Pearson’s correlations among these three environmental parameters and the relative abundances of all associated OTUs were visualized with a correlation network diagram (**Figure 4**).

Fluid pH was positively correlated with the two most abundant OTUs in CSW1.1 (OTU001 and OTU018, classified as Comamonadaceae and Thermoanaerobacterales SRB-2, respectively) and negatively correlated with OTU008 (classified as *Azonexus hydrophilus*), the dominant betaproteobacterial OTU in wells with a pH below 10 (**Table 2** and **Figure 4**). The top OTUs from CSW1.1, OTU001 (classified as Comamonadaceae) and OTU018 (classified as Thermoanaerobacterales SRB-2), were negatively correlated with CO concentration. Except for the betaproteobacterial OTU033, all other OTUs that were positively correlated with CO concentration belonged to the alphaproteobacterial order Sphingomonadaceae (**Figure 4**). Among the OTUs positively correlated with the abundance of methane were Betaproteobacteria OTU004, most abundant in CSW1.2, and Clostridia OTU006, most abundant in CSW1.5 (**Table 2** and **Figure 4**). Additionally, five Gammaproteobacteria OTUs and three Alphaproteobacteria OTUs, two classes known to contain aerobic methanotrophs, were also positively correlated with methane concentration (**Figure 4**). It should be stressed, however, that correlation does not necessarily indicate utilization or production of this compound by the microorganisms.

The most abundant OTUs in CSW1.1, OTU001 (classified as Comamonadaceae) and OTU018 (classified as Thermoanaerobacterales SRB-2), were positively correlated with conductivity, organic acid concentrations, and H_2_ concentrations, and were negatively correlated with ORP (**Table 2**). Two of the most abundant Clostridia OTUs (OTU002 and OTU003), both dominant in QV1.1, were significantly correlated only with well depth (**Table 4**). While **Table 2** and the discussion above denote the sample in which each OTU was most abundant, it should be mentioned that many of those abundant OTUs were found in multiple samples, though at lower abundances (**Supplementary Figure S2**).

**Table 4:** Taxonomy (at the Class level) of metagenomic contigs containing genes-of-interest.

	*mxaF* K14028	*[FeFe]-hyd* K00533	*hyaB* K06281	*hoxH* K00436	*acsB* K14138	*rbcL* K01601	*coxL* K03520	*cooS* K00198	*aprA* K00394	*dsrA* K11180
CSW1.1AC		Clostridia		Beta-pb^∗^		Beta-pb^∗^				
QV1.1A		Clostridia^+^		Beta-pb^∗^ Clostridia^+^	Clostridia^+^	Beta-pb^∗^	Beta-pb^∗∗^	Clostridia^+^	Clostridia^+^	Clostridia
CSW1.3A	Alpha-pb	Clostridia^+^	Beta-pb Clostridia^+^ Alpha-pb Flavobacteria	Beta-pb^∗^ Clostridia^+^ Alpha-pb	Clostridia^+^	Beta-pb^∗^	Beta-pb Alpha-pb	Clostridia^+^	Clostridia^+^	Clostridia
QV1.2A		Clostridia	Alpha-pb Beta-pb Cyanobacteria	Beta-pb		Beta-pb Sphingobacteria Bacilli	Alpha-pb Beta-pb	Clostridia		

*^∗^*Serpentinomonas B1.*^∗∗^*Serpentinomonas A1.*^+^*Dethiobacter alkaliphilus*.*

### Metabolic Potential

**FIGURE 5:**
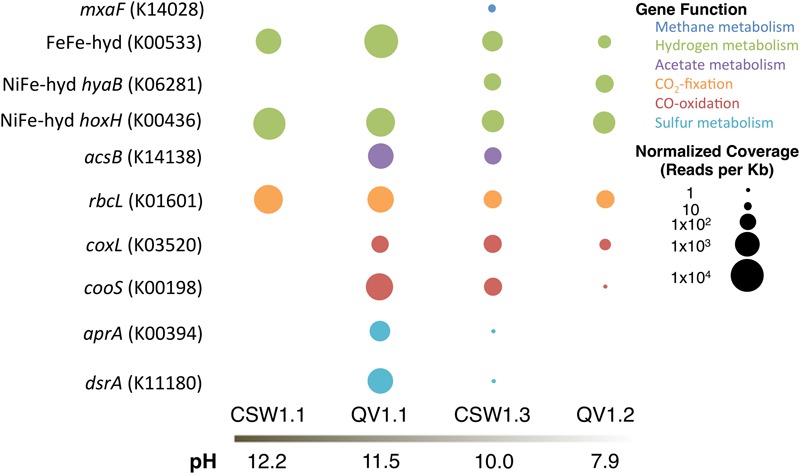
**Relative abundance of protein-encoding genes (with KEGG IDs) in each metagenomic assembly, normalized to reads per Kb and metagenome size**.

To elucidate whether microbes within the serpentinite subsurface environments are capable of metabolizing the geochemical products of serpentinization (specifically hydrogen, methane, acetate, and carbon monoxide) and other environmentally relevant compounds (specifically carbon dioxide and sulfur compounds), assembled and annotated metagenomes from four of the wells were searched for sequences predicted to encode proteins potentially diagnostic of specific metabolic pathways of interest (**Figure 5**). The metagenomes came from samples CSW1.1AC (pH 12.2), QV1.1A (pH 11.5), CSW1.3A (pH 10.1), and QV1.2A (pH 7.9). A summary of the metagenomic assembly statistics can be found in Supplementary Table S2.

To assess the potential for H_2_ metabolism, genes encoding [FeFe]-hydrogenase (KEGG ID: K00533) and [NiFe]-hydrogenases (KEGG ID: K0628, K00436) were sought in the assembled metagenomes. [FeFe]-hydrogenases, often found in fermentative Clostridia species, are typically involved in the production of H_2_ (Vignais et al., 2001; Vignais and Billoud, 2007). [NiFe]-hydrogenases are divided into functional groups: Group 1 includes membrane-bound uptake hydrogenases; Group 2 includes H_2_-sensing hydrogenases; Group 3 includes cytoplasmic hydrogenases catalyzing reversible hydrogen-oxidation; and Group 4 includes archaeal H_2_ production (Vignais et al., 2001; Vignais and Billoud, 2007). The [NiFe]-hydrogenases targeted in this analysis are *hyaB* (K06281), which is a Group 1 (H_2_-oxidizing) hydrogenase, and *hoxH* (K00436), which is a Group 3d (cytoplasmic) hydrogenase.

The [FeFe]-hydrogenase gene was detected in all four metagenomes, but was most abundant in QV1.1A (**Figure 5**). All of the contigs containing the [FeFe]-hydrogenase sequences were classified as Clostridia (**Table 4**). The Group 1 [NiFe]-hydrogenase (*hyaB*) was only detected in the moderate pH CSW1.3A and QV1.2A metagenomes (**Figure 5**), and the contigs that contained the gene were classified as Clostridia, Flavobacteria, Cyanobacteria, and Proteobacteria (Alpha-, and Beta-; **Table 4**). The Group 3d [NiFe]-hydrogenase (*hoxH*), on the other hand, was detected in all four metagenomes (**Figure 5**) and was found on contigs belonging to Betaproteobacteria, Clostridia, and Alphaproteobacteria. The betaproteobacterial contigs it was present on were identified as belonging to *Serpentinomonas* in CSW1.1AC, QV1.1A, and CSW1.3A (**Table 4**).

The metagenomic assemblies were searched for representative genes of bacterial aerobic methane oxidation (particulate methane monooxygenase (*pmoA*; K10944) and methanol dehydrogenase (*mxaF*; K14028) and for a representative gene of archaeal methanogenesis (*mcrA*; K00399). No *pmoA* or *mcrA* genes were identified in any of the metagenomes. CSW1.3A (pH 10.1) contained *mxaF* genes (**Figure 5**) on contigs belonging to members of the Alphaproteobacteria (**Table 4**), likely of the family Methylobacteriaceae, a group of methylotrophs (Lau et al., 2013) found in low abundance in samples from CSW1.3 (**Figure 1**).

The acetyl-coA synthase gene (*acsB*; K14138), suggestive of acetogenesis via the Wood/Ljungdahl pathway (or reductive acetyl-coA pathway; Ragsdale and Pierce, 2008), was only detected in QV1.1A (acetate = 11.2 μM; **Table 1**) and CSW1.3A (acetate < 1.55 μM; **Table 1** and **Figure 5**). All instances of the contigs containing the gene were classified as being closely related to the clostridium *Dethiobacter alkaliphilus* (**Table 4**), which makes up 11 and 25% of the QV1.1A and CSW1.3A communities, respectively (**Figure 1**).

The high pH of the serpentinite environment limits the availability of CO_2_, potentially leading to the use of CO as both an electron donor and an inorganic carbon source. To assess CO metabolism at CROMO, two forms of CO dehydrogenase were sought in the metagenomes; *coxL* (K03520) and *cooS* (K00198) were used to identify aerobic and anaerobic CO oxidation, respectively. Neither gene was detected in CSW1.1AC, and both were most abundant in QV1.1A (CO = 0.142 μM; **Table 1** and **Figure 5**). All of the *cooS* genes detected were on contigs that belonged to Clostridia, while the *coxL* genes were more diverse, belonging to contigs identified as Betaproteobacteria and Alphaproteobacteria in the moderate to circumneutral wells, and to the Betaproteobacteria closely related to *Serpentinomonas* strain A1 in the high pH wells (**Table 4**; Suzuki et al., 2014).

Sequences encoding the *rbcL* gene (K01601) of the RuBisCo enzyme used in the Calvin–Benson–Bassham cycle were detected in all four metagenomes (**Figure 5**). In CSW1.1AC and QV1.1A, *rbcL* genes were found on contigs with high similarity to *Serpentinomonas* strain B1 (**Table 4**; Suzuki et al., 2014). The sequences detected in CSW1.3A also belonged to Betaproteobacteria, but of the family Burkholderiaceae (unlike the Comamonadaceae found in the higher pH wells), and the *rbcL* sequences from QV1.2A were more diverse, identified on contigs as coming from Sphingobacteria and Bacilli, as well as Betaproteobacteria (**Table 4**).

The potential metabolism of sulfur compounds was assessed by searching for dissimilatory sulfite reductase (*dsrA*; K11180) and dissimilatory adenosine-5′-phosphosulfate reductase (*aprA*; K00394). Both genes were detected at high abundance in QV1.1A and at low abundance in CSW1.3A (**Figure 5**). In both QV1.1A and CSW1.3A the contigs containing *aprA* and *dsrA* were classified as Clostridia (**Table 4**), and both of these samples contained a relatively high abundance of *Dethiobacter* sp. and SRB-2 (**Figure 1**). In contrast, neither gene was detected in wells CSW1.1AC or QV1.2A.

## Discussion

### Serpentinite Environment at CROMO

The geochemical variability among multiple wells at CROMO allowed us to distinguish groundwater with varying degrees of influence from subsurface serpentinization processes. For example, the shallow CSW1.4 and QV1.2 wells produced pH ∼8 water that had comparatively high levels of DO, DIC, and ORP (**Table 1**), which is consistent with oxygenated water that was recently exposed to the surface and was not noticeably affected by serpentinization. In contrast, the CSW1.1 well (pH 12.2) exhibited high conductivity and high concentrations of H_2_ and small organic molecules (**Table 1**), consistent with water heavily influenced by subsurface serpentinization-associated reactions. Furthermore, CSW1.1 has remarkably low bacterial diversity, suggesting that it represents the most extreme window into the serpentinite subsurface. Other wells have weaker serpentinization signatures, such as that of CSW1.3, which is almost as deep as CSW1.1 and has almost as much H_2_, but its water is pH ∼10, suggesting a dilution of serpentinization-influenced water with shallower, surface-influenced water.

It should be noted that all of the wells, save CSW1.1 and QV1.1, were cased with PVC pipe upon drilling, essentially isolating the flow of water into the wells (Cardace et al., 2013). The larger diameter CSW1.1 and QV1.1 wells remained uncased, and therefore, could experience some fluid input from above the drilled depth, which suggests that true serpentinite end-member fluids could be even more extreme than those measured here.

Bacterial diversity trends across the CROMO wells are remarkably consistent with the geochemistry of the wells. The overall bacterial community compositions of water from the most serpentinization-influenced (CSW1.1 and QV1.1) wells are tightly correlated with pH, organic acid concentrations, and low ORP values (**Figure 3**). Conversely, the community compositions of shallow, pH 7.8–9 wells (CSW1.2, CSW1.4 and QV1.2) are more tightly coupled to higher ORP, DO, and DIC, which is consistent with the interpretation of geochemistry data that these wells represent mixing of surface waters and serpentinization-influenced groundwater.

The distributions of individual genes, as measured by metagenomic sequencing, are also consistent with these biogeochemical trends. For example, genes involved in CO oxidation were completely absent in the well (CSW1.1) with the lowest concentration of CO, and these genes were present in each of the other three metagenomes (**Table 1** and **Figure 5**). Genes associated with methane metabolism were surprisingly rare in the CROMO metagenomes, but one gene involved in bacterial methane oxidation (*mxaF*) was found in CSW1.3, which contains higher methane concentrations than the other three wells with metagenomes (**Table 1** and **Figure 5**).

### Betaproteobacteria

*Serpentinomonas* is a newly proposed genus within family Comamonadaceae currently represented by three strains isolated from the Cedars, another site of subsurface serpentinization in northern California (Suzuki et al., 2014). An OTU with 100% sequence identity to *Serpentinomonas* B1 comprised over 50% of environmental 16S rRNA gene sequences from CSW1.1 (pH 12.2) and over 25% of sequences from QV1.1 (pH 11.5) and has been detected in CROMO wells at other time points (Crespo-Medina et al., 2014). The abundance of this OTU was positively correlated with pH, H_2_, conductivity, and organic acids and negatively correlated with ORP and CO (**Figure 1** and **Table 2**).

Each strain of S*erpentinomonas* (A1, B1, and H1) from the Cedars contains slightly different genes with regards to nitrate reduction, hydrogen oxidation, and carbon fixation (Suzuki et al., 2014). All strains are capable of CO_2_-fixation via the Calvin–Benson–Bassham cycle and contain the *rbcL* gene coding for the RuBisCo enzyme, which was detected in all four metagenomes from CROMO wells and was most abundant in CSW1.1 (**Figure 5**). The *coxL* gene (involved in aerobic CO oxidation) is found only in *Serpentinomonas* A1 and was detected in QV1.1, but not CSW1.1 in this study, suggesting different phylotypes of the abundant organism in different wells (**Figure 5** and **Table 4**). Only the H1 strain of *Serpentinomonas* contains the Group 1 [NiFe]-hydrogenase hydrogen-oxidation gene *hyaB*, which was not detected in either of the wells with extreme pH (**Figure 5**). However, Group 3d [NiFe]-hydrogenases, which are cytoplasmic hydrogenases capable of reversible H_2_-oxidation, are found in all three strains of *Serpentinomonas* (Suzuki et al., 2014) and were detected in all four CROMO wells (**Figure 5**). *Serpentinomonas* strains containing Group 3d (and not Group 1) [NiFe]-hydrogenase genes were experimentally shown to oxidize hydrogen to support autotrophic growth, so it has been speculated that Group 3d [NiFe]-hydrogenase genes, such as *hoxH*, might be the key to H_2_ metabolism in these organisms (Suzuki et al., 2014). None of the *Serpentinomonas* strains contain *ACS, cooS, aprA*, or *dsrA* (Suzuki et al., 2014), and none of those genes were detected in CSW1.1, where *Serpentinomonas* dominated the community (**Figures 1, 5**). These data support interpretations (Schrenk et al., 2013) that *Serpentinomonas*-like organisms are hydrogen-oxidizing, carbon-fixing members of serpentinite-hosted ecosystems, able to persist at extreme pH and thrive on the chemical disequilibrium of the mixing zone between anoxic end-member and oxygenated surface fluids.

The most abundant betaproteobacterial OTU found in QV1.2 (pH 7.9) was OTU008, which made up 33.1 ± 6.8% of the total community and exhibited 100% sequence identity to *Azonexus hydrophilus* (family Rhodocyclaceae; **Table 2** and **Figure 1**). This organism is a motile, non-spore-forming aerobe isolated from freshwater springs in Taiwan and Korea (Chou et al., 2008), and it has a circumneutral optimal growth pH. In this study, OTU004 exhibited a negative correlation with pH, which is consistent with a circumneutral optimal growth pH. It has been detected in sewage treatment facilities (Auguet et al., 2015; Yan et al., 2015) and coal bed methane environments (Guo et al., 2012), as well as in the deep subsurface (Ise et al., 2016; Jangir et al., 2016). In QV1.2A, Group 1 [NiFe]-hydrogenase gene *hyaB* and the *rbcL* gene of RuBisCo were detected on contigs identified as *Azonexus hydrophilus* (Supplementary Dataset S1), suggesting that these Betaproteobacteria are involved in H_2_-oxidation and carbon fixation in the moderate wells.

*Alicycliphilus denitrificans* made up 32.9% of CSW1.2 (pH 9.3), making it the most abundant betaproteobacterial OTU in the sample (**Figure 1** and **Table 2**). This organism is a facultative denitrifying bacterium that can use acetate as a carbon source (Mechichi et al., 2003). OTU004 (classified as *A. denitrificans*) was positively correlated with methane (**Table 2**), but this organism is not known to be involved in methane cycling.

### Clostridia

Clostridia have been detected at sites of continental serpentinization around the world (Brazelton et al., 2012, 2013; Suzuki et al., 2013; Tiago and Veríssimo, 2013; Woycheese et al., 2015), where they are thought to inhabit the anoxic end-member serpentinite fluids (Schrenk et al., 2013). An abundance of [FeFe]-hydrogenases belonging to Clostridia in metagenomic data from serpentinite springs (Brazelton et al., 2012) suggests that these abundant organisms are producing H_2_, but additional clues to their physiology and ecology are lacking.

Operational taxonomic units classified as Clostridia were very abundant in the high pH wells at CROMO. The clostridial OTUs at CROMO were either classified as *Dethiobacter*, Thermoanaerobacterales SRB-2, or unclassified Clostridia. The most extreme well, CSW1.1 (pH 12.2), contained 13.9 ± 6.5% Clostridia, mostly represented by an OTU classified as SRB-2. QV1.1 (pH 11.5) displayed a greater abundance and diversity of Clostridia, as well as a shift in clostridial community composition between field replicates (**Figure 1**). The deeper QV1.1 samples (field replicates A/B) had more SRB-2 and fewer *Dethiobacter* than in waters naturally found higher up in the well (i.e., filtered later as water was drawn down).

*Dethiobacter alkaliphilus* is an anaerobic alkaliphile that was originally isolated from a Mongolian soda lake; it utilizes H_2_ as an electron donor, sulfur compounds (S°, thiosulfate, polysulfide) as electron acceptors, and acetate as a carbon source (Sorokin et al., 2008). *Dethiobacter* has been detected at sites of continental serpentinization and is hypothesized to live in the deep, anoxic serpentinite end-member fluids (Brazelton et al., 2012; Suzuki et al., 2013; Tiago and Veríssimo, 2013; Crespo-Medina et al., 2014; Woycheese et al., 2015). Crespo-Medina et al. (2014) detected growth in CROMO microcosms dominated by *D. alkaliphilus* with H_2_-headspace and the addition of thiosulfate with either acetate or methane as a carbon source. It should be noted that since there is no known mechanism for Clostridia to consume methane, it is believed there was a cryptic organism within the microcosms making methane-derived carbon bioavailable to *Dethiobacter* (Crespo-Medina et al., 2014). In the QV1.1A metagenome, the genes attributed to *Dethiobacter* are *hoxH* (H_2_ sensing), FeFe-hydrogenase (H_2_-production), *acsB* (acetogenesis), and *cooS* (anaerobic CO-oxidation) (**Table 4**). These data suggest that the abundant *Dethiobacter* encode several metabolic pathways that might allow them to adapt to changing conditions in the subsurface or in the well water.

Members of the family Thermoanaerobacterales, identified as belonging to the SRB-2 lineage, were abundant in CSW1.1 and QV1.1 (**Figure 1**). The SRB-2 OTUs share high sequence identity with clones from CVA fluids and the Cedars (**Table 2**; Suzuki et al., 2013; Tiago and Veríssimo, 2013). Not much is known about this uncultivated group, but the Thermoanaerobacterales include organisms capable of sulfate reduction (Pereira et al., 2011), fermentative acetate oxidation (Oehler et al., 2012), fermentative hydrogen production (Rittmann and Herwig, 2012), and homoacetogenesis (Ljungdahl, 1994). The [FeFe]-hydrogenase and *acsB* genes detected in the metagenomes could not be classified at the family level (with some exceptions, see **Table 4**) but they were all classified as Clostridia, consistent with SRB-2 and perhaps other unclassified Clostridia as potentially acetogenic, sulfur-reducing alkaliphiles in the subsurface serpentinite fluids.

Sequences belonging to Erysipelotrichia (another class of the phylum Firmicutes) were inferred to represent the most likely inhabitants of serpentinite end-member fluids exiting from springs at the Tablelands, Newfoundland (Brazelton et al., 2013). An OTU classified as genus *Erysipelothrix* was found in three moderately high pH CROMO wells, but in none of the other wells (**Figure 1**). None of the protein-coding genes in the CROMO metagenomes were classified as *Erysipelothrix* (**Table 4**).

### Other Community Members

All of the OTUs in CSW1.1 and QV1.1 that made up greater than 1% of sequences in either well belonged to the Betaproteobacteria or Clostridia. Furthermore, all of the metagenomic contigs containing key genes of interest from these two wells were classified as one of these two classes of bacteria (**Table 4**). The moderately high pH and circumneutral wells exhibited slightly higher diversity in not only 16S rRNA genes (**Figure 1**), but also in the variety and taxonomic classification of protein-encoding genes (**Figure 5** and **Table 4**). Additional bacterial taxa in these wells included Alphaproteobacteria, Deltaproteobacteria, and Bacteroidetes. These results suggest that a breadth of organisms are capable of using products of serpentinization, but might be restricted by the extreme pH conditions in wells containing a greater degree of end-member fluids.

It is notable that archaea were absent from all CROMO 16S rRNA sequences and were very rare (1% or less of sequences) in the metagenomic data (**Table 3**). Studies of other sites of continental serpentinization, in contrast, have detected methanogenic Euryarchaeota (Suzuki et al., 2013; Tiago and Veríssimo, 2013). Methane isotopologue analyses have suggested that the methane at CROMO displays a thermogenic signature, while methane from the nearby Cedars site (from which *Serpentinomonas* was isolated) has a more microbial signature (Wang et al., 2015). These isotope geochemistry results are consistent with the lack of methanogens in CROMO wells. Additionally, OD-1 and Chloroflexi, both of which are bacteria that Suzuki et al. (2013) hypothesized to be endemic to end-member serpentinite fluids, were not detected in any of the CROMO fluids. Therefore, the subsurface fluids in CROMO wells appear to be an unfavorable environment for several microorganisms that are abundant in other sites of serpentinization.

## Conclusion

By studying groundwater with a range of geochemical characteristics, we were able to identify the bacterial taxa with the strongest correlations to the environmental variables (such as pH, CO, and CH_4_) indicative of subsurface serpentinization processes. These data suggest that various Clostridia taxa are potentially capable of anaerobic CO-oxidation, acetogenesis, and the reduction of sulfur compounds in extremely high pH, anoxic subsurface fluids heavily influenced by serpentinization, while microaerophilic *Serpentinomonas* (Betaproteobacteria) are capable of H_2_-oxidation and CO_2_-fixation (and in some cases, aerobic CO-oxidation) in mixing zones where deep, anoxic fluids interact with oxygenated surface waters. These data provide tractable targets for further biogeochemical and microbiological analyses of serpentinite-hosted microbial ecosystems at CROMO and elsewhere.

The results of this study support those of other recent studies of serpentinite-hosted ecosystems that have identified a few key bacterial taxa that are common to such systems. Furthermore, our results significantly expand our current understanding of the microbial ecology of subsurface, serpentinite-hosted ecosystems. Previously, studies of the microbiology of continental serpentinites have focused on opportunistic sampling from a small number of surface-exposed sites (Brazelton et al., 2012; Suzuki et al., 2013; Tiago and Veríssimo, 2013). Because these studies must sample the surface expression of subsurface processes, identifications of taxa that are truly endemic to the subsurface must be indirect inferences that rely on assumptions of hydrology and sampling methodology. By directly accessing the serpentinite subsurface using wells at CROMO, we were able to observe subsurface microbial communities that had not directly experienced the surface processes that influence the composition of microbial communities sampled from springs. Furthermore, access to the groundwater revealed the absence of several taxa that were identified as key members of other serpentinite-hosted ecosystems, which highlights the remarkably low diversity of organisms apparently capable of thriving in these high pH subsurface fluids.

## Author Contributions

KT, WB, and MS designed the study. WB, MK, and DC performed field sampling. KT, WB, and AH performed the bioinformatic analyses and interpretations. KT and WB performed statistical analyses. MK, DC, TH, and TM contributed to the geochemical analyses and interpretations. KT, WB, MK, AH, DC, TH, TM, and MS wrote the manuscript.

## Conflict of Interest Statement

The authors declare that the research was conducted in the absence of any commercial or financial relationships that could be construed as a potential conflict of interest.

## Footnotes

1 https://nai.nasa.gov/research/data-sharing/

2 www.img.jgi.doe.gov

## Supplementary Material

FIGURE S1 **Microbial community structure. (A)** Rarefaction analysis of 16S rRNA amplicon sequences. Multiple samples from the same well represent field replicates. **(B)** Community similarity dendrogram calculated from Bray-Curtis index. Samples connected by red lines are not distinguishable from one another by a SIMPROF test and ANOSIM analysis indicated that there is a significant difference in community composition between wells (*R* = 0.9, *p*-value < 0.05).

FIGURE S2 **Heatmap of most abundant OTUs and geochemical parameters across all samples.** Dendrogram at the top represents community similarity between samples and red lines indicate no statistical difference between field replicates, as determined by SIMPROF (Clarke, 1993).
