# Supplementary figures and images for: Serpentinization-Influenced Groundwater Harbors Extremely Low Diversity Microbial Communities Adapted to High pH
Source: Front Microbiol. 2017 Mar 1;8:308. doi: 10.3389/fmicb.2017.00308 (PMC5331062; doi:10.3389/fmicb.2017.00308)

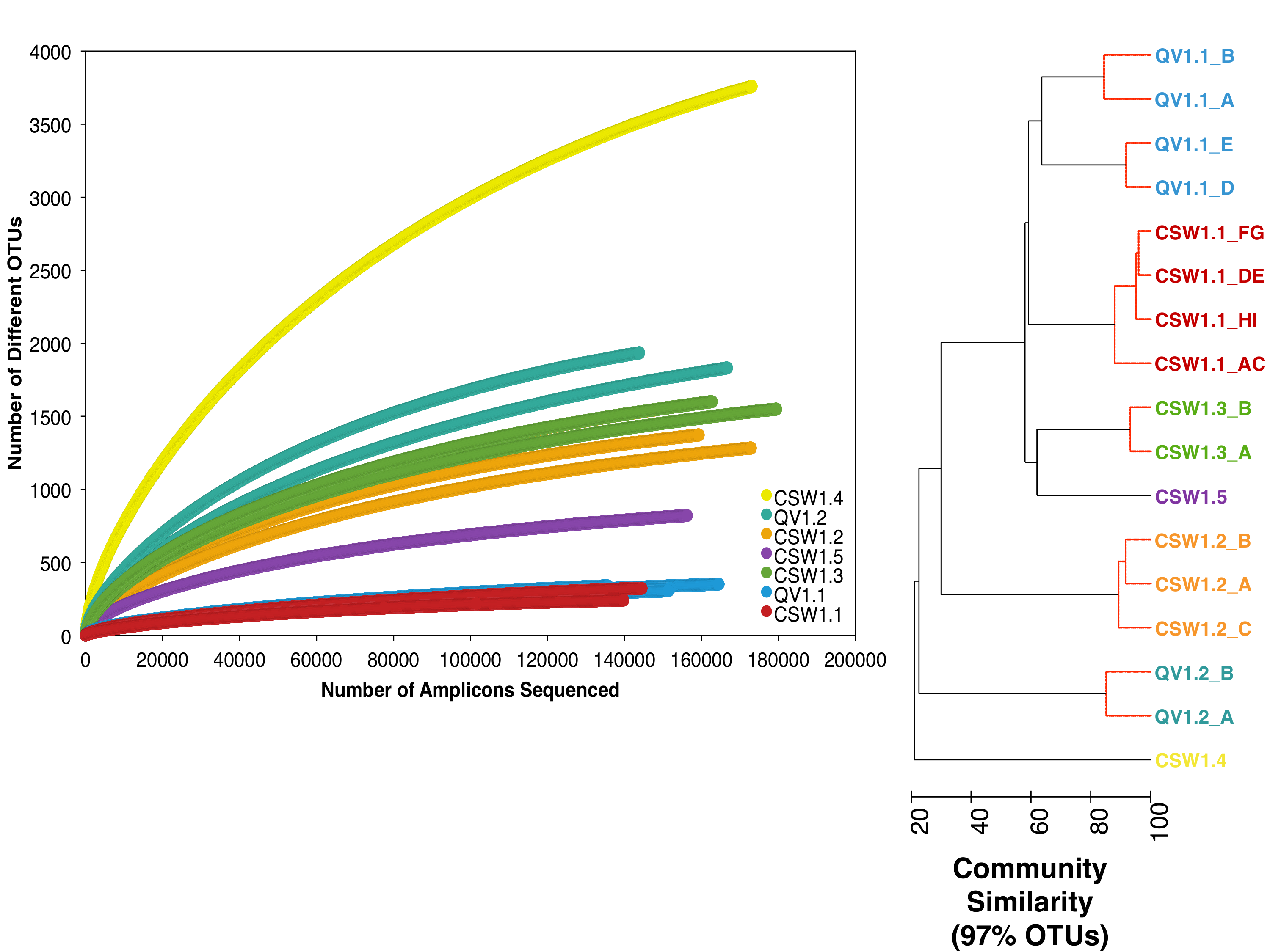

Supplement: FIGURE S1 — Microbial community structure. (A) Rarefaction analysis of 16S rRNA amplicon sequences. Multiple samples from the same well represent field replicates. (B) Community similarity dendrogram calculated from Bray-Curtis index. Samples connected by red lines are not distinguishable from one another by a SIMPROF test and ANOSIM analysis indicated that there is a significant difference in community composition between wells (R = 0.9, p-value < 0.05). [file Image_1.TIF]

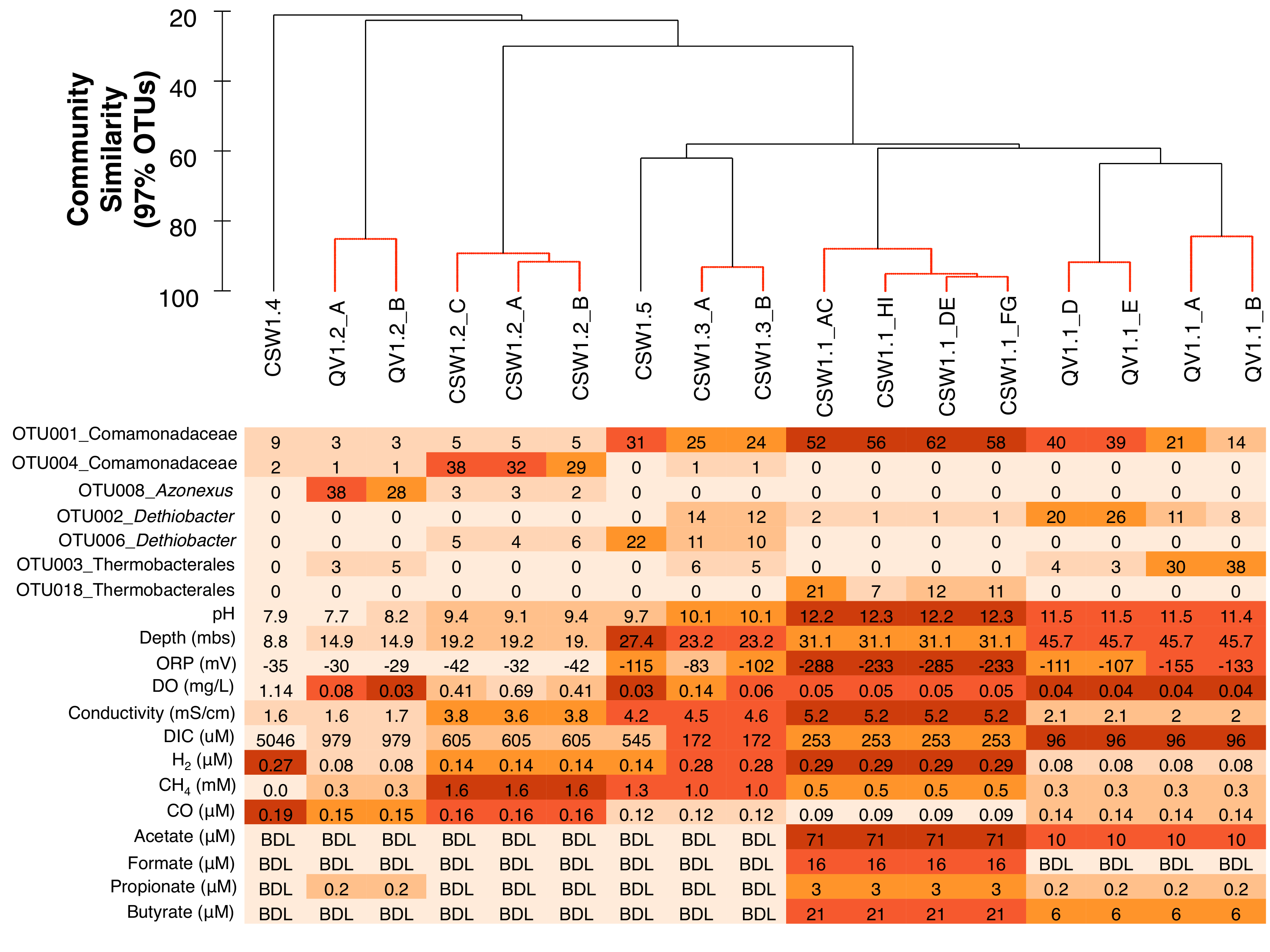

Supplement: FIGURE S2 — Heatmap of most abundant OTUs and geochemical parameters across all samples. Dendrogram at the top represents community similarity between samples and red lines indicate no statistical difference between field replicates, as determined by SIMPROF (Clarke, 1993). [file Image_2.TIF]
