# Supplementary material for: Serpentinization-Influenced Groundwater Harbors Extremely Low Diversity Microbial Communities Adapted to High pH
Source: Front Microbiol. 2017 Mar 1;8:308. doi: 10.3389/fmicb.2017.00308 (PMC5331062; doi:10.3389/fmicb.2017.00308)
Supplement: Supplementary file 4 [file Table_1.DOCX]

Supplementary Material

**Serpentinization-influenced groundwater harbors extremely low diversity microbial communities adapted to high pH**

Katrina I. Twing^*^, William J. Brazelton, Michael D. Kubo, Alex J. Hyer, Dawn Cardace, Tori M. Hoehler, Tom M. McCollom, and Matthew O. Schrenk

*** Correspondence:** Katrina I. Twing: Katrina.twing@utah.edu

**Supplementary Table 1.** Significant pairwise Pearson’s correlations between environmental parameters.

|  |  | **Correlation** | **R** | **p-value** |
| --- | --- | --- | --- | --- |
| Depth | pH | + | 0.79 | < 0.001 |
|  | DO | - | 0.59 | 0.01 |
|  | DIC | - | 0.59 | 0.01 |
| Temp | H_2_ | - | 0.54 | 0.02 |
| pH | Butyrate | + | 0.77 | < 0.001 |
|  | Formate | + | 0.73 | < 0.001 |
|  | Propionate | + | 0.66 | 0.004 |
|  | Acetate | + | 0.64 | 0.005 |
|  | ORP | - | 0.87 | < 0.001 |
|  | CO | - | 0.75 | < 0.001 |
|  | DIC | - | 0.58 | 0.01 |
|  | DO | - | 0.55 | 0.02 |
| ORP | CO | + | 0.87 | < 0.001 |
|  | Butyrate | - | 0.94 | < 0.001 |
|  | Acetate | - | 0.92 | < 0.001 |
|  | Propionate | - | 0.89 | < 0.001 |
|  | Formate | - | 0.88 | 0.03 |
|  | Conductivity | - | 0.63 | < 0.001 |
|  | H_2_ | - | 0.53 | 0.006 |
| DO | DIC | + | 0.80 | < 0.001 |
|  | CO | + | 0.65 | 0.004 |
| Conductivity | H_2_ | + | 0.77 | < 0.001 |
|  | Formate | + | 0.69 | 0.002 |
|  | Propionate | + | 0.67 | 0.003 |
|  | Acetate | + | 0.66 | 0.004 |
|  | Butyrate | + | 0.59 | 0.01 |
|  | CO | - | 0.80 | < 0.001 |
| DIC | CO | + | 0.57 | 0.02 |
| H_2_ | Formate | + | 0.66 | 0.004 |
|  | Propionate | + | 0.64 | 0.005 |
|  | Acetate | + | 0.61 | 0.009 |
|  | Butyrate | + | 0.55 | 0.02 |
|  | CO | - | 0.60 | 0.01 |
| CO | Acetate | - | 0.82 | < 0.001 |
|  | Formate | - | 0.81 | < 0.001 |
|  | Propionate | - | 0.81 | < 0.001 |
|  | Butyrate | - | 0.80 | < 0.001 |
| Formate | Acetate | + | 0.99 | < 0.001 |
|  | Propionate | + | 0.99 | < 0.001 |
|  | Butyrate | + | 0.97 | < 0.001 |
| Acetate | Propionate | + | 0.99 | < 0.001 |
|  | Butyrate | + | 0.99 | < 0.001 |
| Propionate | Butyrate | + | 0.98 | < 0.001 |
