# Supplementary material for: Serpentinization-Influenced Groundwater Harbors Extremely Low Diversity Microbial Communities Adapted to High pH
Source: Front Microbiol. 2017 Mar 1;8:308. doi: 10.3389/fmicb.2017.00308 (PMC5331062; doi:10.3389/fmicb.2017.00308)
Supplement: Supplementary file 5 [file Table_2.DOCX]

Supplementary Material

**Serpentinization-influenced groundwater harbors extremely low diversity microbial communities adapted to high pH**

Katrina I. Twing^*^, William J. Brazelton, Michael D. Kubo, Alex J. Hyer, Dawn Cardace, Tori M. Hoehler, Tom M. McCollom, and Matthew O. Schrenk

*** Correspondence:** Katrina I. Twing: Katrina.twing@utah.edu

**Supplementary Table 2.** Metagenomic assembly statistics.

|  | **Metagenome size**  **(bp)** | **N50**  **(bp)** | **Number of contigs**  **(≥0 bp)** | **Number of contigs**  **(>500 bp)** | **Number of contigs**  **(≥25,000 bp)** | **Total length of contigs**  **(≥0 bp)** | **Total length of contigs**  **(>500 bp)** | **Total length of contigs**  **(≥25,000 bp)** |
| --- | --- | --- | --- | --- | --- | --- | --- | --- |
| CSW1.1AC | 7,120,514 | 5,164 | 4,286 | 2,528 | 20 | 7,694.792 | 81,613 | 2,130,628 |
| QV1.1A | 16,449,643 | 16,993 | 9,521 | 4,007 | 113 | 18,218,553 | 445,690 | 7,267,849 |
| CSW1.3A | 60,810,993 | 12,310 | 55,893 | 17,058 | 396 | 73,247.524 | 445,699 | 22,489,625 |
| QV1.2A | 67,696,686 | 2,747 | 135,127 | 39,147 | 123 | 99,192,681 | 669,886 | 12,807,872 |
